# A multilevel examination of gender differences in the association between features of the school environment and physical activity among a sample of grades 9 to 12 students in Ontario, Canada

**DOI:** 10.1186/1471-2458-12-74

**Published:** 2012-01-24

**Authors:** Erin P Hobin, Scott T Leatherdale, Steve Manske, Joel A Dubin, Susan Elliott, Paul Veugelers

**Affiliations:** 1 School of Public Health and Health Systems, University of Waterloo, Waterloo, Ontario N2L 3G1, Canada; 2 Propel Centre for Population Health Impact, University of Waterloo, Waterloo, Ontario N2L 3G1, Canada; 3 Department of Statistics and Actuarial Science, University of Waterloo, Waterloo, Ontario N2L 3G1, Canada; 4 Faculty of Applied Health Sciences, University of Waterloo, Waterloo, ON N2L 3G1, Canada; 5 School of Public Health, University of Alberta, Edmonton, Alberta T6G 2T4, Canada

**Keywords:** Physical activity, Adolescents, Gender, Environment, Prevention, School

## Abstract

**Background:**

Creating school environments that support student physical activity (PA) is a key recommendation of policy-makers to increase youth PA. Given males are more active than females at all ages, it has been suggested that investigating gender differences in the features of the environment that associate with PA may help to inform gender-focused PA interventions and reduce the gender disparity in PA. The purpose of this cross-sectional study was to explore gender differences in the association between factors of the school environment and students' time spent in PA.

**Methods:**

Among a sample of 10781 female and 10973 male students in grades 9 to 12 from 76 secondary schools in Ontario, Canada, student- and school-level survey PA data were collected and supplemented with GIS-derived measures of the built environment within 1-km buffers of the 76 schools.

**Results:**

Findings from the present study revealed significant differences in the time male and female students spent in PA as well as in some of the school- and student-level factors associated with PA. Results of the gender-specific multilevel analyses indicate schools should consider providing an alternate room for PA, especially for providing flexibility activities directed at female students. Schools should also consider offering daily physical education programming to male students in senior grades and providing PA promotion initiatives targeting obese male students.

**Conclusions:**

Although most variation in male and female students' time spent in PA lies between students within schools, there is sufficient between-school variation to be of interest to practitioners and policy-makers. More research investigating gender differentials in environment factors associated with youth PA are warranted.

## Background

Adolescence is a particularly important developmental stage as not only can lifestyle choices impact health and sense of well-being in the short-term, but they also can affect adult onset of chronic diseases including obesity, type 2 diabetes, and some cancers [1,2]. Unfortunately, direct measures of physical activity (PA) recently collected in both the US and Canada suggest the vast majority of young people do meet the recommended 60 minutes dose of PA required for adequate growth and health [3,4]. For example, among Canadians aged 6 to 19 years, objective measures indicate only 9% of males and 4% of females meet the 60 minutes per day recommendation for PA, with twice as many 6 to 10 year olds meeting this criterion as 15 to 19 year olds [4].

Given there is a well-established tendency for males to be more active than females at all ages, recent discussions suggest that gender differences should be considered within population-level interventions designed to increase PA [5,6]. Population-level interventions that modify environment level factors are believed to be important for creating more supportive environments for PA and benefiting the PA levels of everyone exposed to this environment to some degree [7]. By affecting large numbers of people, not just individuals enrolled in a particular intervention, these environment-level changes can potentially have broad-based and long-lasting impact on PA [8,9]. Consequently, identifying the unique role environments can play in male and female adolescent PA promotion may serve as a critical component to increase PA among this population, decrease the discrepancy between male and female adolescent PA at the population level, and inform PA gender-focused intervention planning [6,10].

Since students in Canada spend almost 200 days per year in school, schools are a key environment for large-scale population-level PA initiatives among adolescents. The creation of a supportive school environment is believed to have enormous potential to encourage more PA by providing opportunities and cues that can facilitate PA [11]. Indeed, the Canadian Federal/Provincial/Territorial Framework for Action to Promote Healthy Weights recommended making schools' social and built environments more supportive of PA by providing students with school physical education (PE), PA programming, and PA-related facilities whenever possible [12]. Many studies examining student- and school-level characteristics associated with student PA have found positive relationships with school PE and PA programming [13-16].

Results from recent research investigating factors of the school built environment also suggest gender discrepancies in the influence of school PA-related facilities on student PA. For example, results of a study investigating the number of PA facilities within walking distance of school and after-school PA behaviour among 1394 12^th ^grade females attending 22 secondary schools in the US found females attending schools with ≥ 5 facilities within a 1-km buffer reported more PA per day than females in schools with < 5 facilities [17]. Similar results were noted in a sample of 16471 students in grades 8 to 10 attending 68 schools in Norway. Students in this study who attended a school with eight PA-related facilities on school grounds engaged in almost three times more PA during school recess compared with students attending a school with the lowest number of facilities [18]; however, gender differences were detected in the particular types of school PA-related facilities associated with student PA. The PA levels of male students were higher among those attending schools with a soccer field, playground equipment, sledding hill, or an area for hopscotch or skipping compared to males attending schools without these facilities [18]. Access to a sledding hill at school was the only school facility to influence the PA of females [18]. Other than areas for hopscotch and skipping, however, this study did not examine school PA facilities that may be more attractive to female students, such as aerobic or dance studios. Indeed, observational research suggests more traditional school PA facilities, such as soccer fields and playground equipment, are predominantly used by males for sports and other physical activities, with females remaining passive and not participating [19].

Consideration of gender-specific needs in school PE and PA program planning as well as the design of school PA-related facilities may help alleviate the gender discrepancies in PA evident among adolescents. With the scarcity of gender studies focused on environmental factors associated with adolescent PA, the present study aims to extend previous research by determining gender differences in the association between factors of the schools' social (i.e., school PE and PA programming) and built environments and students' time spent in PA among a sample of secondary school students in Ontario, Canada.

## Methods

### Design

This secondary analysis examined cross-sectional self-report data collected from students in grades 9 to 12 and administrators at 76 high schools in Ontario as part of the SHAPES-Ontario study (2005-2006). Objective measures of the built environment surrounding each of the 76 schools were also collected. The University of Waterloo Office of Research Ethics and appropriate School Board Ethics committees approved this study and data collection procedures.

#### Data sources and procedures

##### Student-level data

Student-level data were collected from consenting students using the SHAPES student-level PA module with validated measures for student PA and BMI [20]. The SHAPES student-level PA module asks students about their demographic information and PA-related behaviours. Testing of the SHAPES student-level PA module with students in grades 9 to 12 demonstrated satisfactory criteria validity, test retest reliability, and comprehension of the survey [20]. Most notably, Spearman correlations between the self-reported PA measures and directly measured PA with accelerometers were significant (r = 0.44) and consistent with other validated tools among youth [20]. Moreover, correlations between self-reported body mass index (BMI) and measured height and weight were high (Spearman r = 0.90) and significant (*p *< 0.001). Classification of weight status by BMI was similar using self-reported values compared to measured values [20]. Additional details about SHAPES, SHAPES-Ontario, and the survey measures and their psychometric properties are available in print [20,21] and online http://www.shapes.uwaterloo.ca. All student-level surveys were completed in class time and participants were not provided compensation. Actively providing information to parents with passive permission was used to reduce demands on schools and to increase student participation rates.

##### Environment-level data

As part of the SHAPES-Ontario project, all 76 school administrators completed the Canadian Lifestyle and Fitness Research Institute's School Capacity Survey. Administrators indicated the availability of 14 different PA-related facilities on school grounds as well as the geographical location of the school (i.e., urban, suburban, rural). Researchers mailed administrators a standardized package including a consent form and the School Capacity Survey. If a school did not return a completed survey within 4-weeks, researchers emailed a standardized reminder to the school administrator.

Seven built environment features in the neighbourhood surrounding the 76 schools were assessed using geographic information systems (GIS) data from the Desktop Mapping Technologies Inc (DMTI) data resource, including destinations of interest to adolescents (e.g., parks, shopping malls) and neighbourhood design features. Neighbourhood built environment data within 1-km circular buffers surrounding each of the 76 schools were provided by two DMTI Spatial resources; the CanMap RouteLogistics (CMRL) spatial information database and the Enhanced Points of Interest (EPOI) database. Consistent with previous research [22,23], the process of identifying and linking the DMTI-EPOI data to the SHAPES-Ontario student and school survey data involved three steps: 1) geocoding the address for each SHAPES Ontario school; 2) creating 1 km circular buffers (i.e., bounded areas surrounding each school in which the built environment structures were quantified); and, 3) linking quantified built environment data for each school to the student and school survey data. Arcview 3.3 (ESRI, 2002) software was used to geocode the school addresses and to create the 1 km buffers.

School neighbourhood SES information was collected from the 2006 Canadian Census Tract Profiles [24] by entering the postal codes of the schools.

### Measures

#### Students' time spent in physical activity

To be consistent with Canada's Physical Activity Guidelines for youth aged 12 to 17 years, this study defines student PA as average daily minutes spent performing moderate to vigorous PA (MVPA) [25]. To calculate MVPA, each student's responses to the questions "Mark how many minutes of moderate physical activity you did on each of the last 7 days" and "Mark how many minutes of hard physical activity you did on each of the last 7 days" were summed and divided by 7 days. Moderate PA was defined as "lower intensity physical activities such as walking, biking to school, and recreational swimming." Hard PA was defined as "jogging, team sports, fast dancing, jump-rope, and any other physical activities that increase your heart rate and make you breathe hard and sweat." Responses were provided by indicating the number of hours (0-4 h) and 15-min increments (0-45 min) that each type of PA was performed for each day of the previous week.

#### Student characteristics

Students were asked to report their age, grade, gender, and height and weight. Age- and sex-adjusted body mass index (BMI) cut-points derived from the WHO growth charts were used to classify students' weight status [26].

Student-level predictors were consistent with previous research [15,27,28]. Student respondents reported how they usually got to and from school in the last 7 days [active, mixed, inactive (referent)], if they were enrolled in PE [Participated in 1-5 days of PE in past 7 days/Participated in 0 days of PE in past 7 days], and if they participated in school intramural activities or varsity sports teams [Yes/No (referent)].

Finally, students were asked to report on their participation in activities for flexibility and strength. To assess their participation in flexibility-related activities, students responded to the single item: "In the last 7 days, how many days did you do exercises for flexibility, such as stretching or yoga" with response options from 0 to 7 days. Similarly, to assess their participation in strength-related activities, students responded to the single item: "In the last 7 days, how many days did you do exercises to strengthen or tone your muscles, such as push-ups, sit-ups, yoga, or weight lifting" with response options from 0 to 7 days. Consistent with Canada's PA Guidelines for youth, responses for participation in activities for flexibility and strength were classified as "3 or more days per week" or "less than 3 days per week" [25,29].

#### School social environment variables

School-level predictors were also consistent with previous research [15,28]. Administrators reported if their school offers daily PE class [5 days of PE class per week/< 5 days of PE class per week (referent)], intramural PA programs [Yes/No (referent)], and varsity sports teams [Yes/No (referent)].

#### School and neighbourhood built environment variables

For this study, measures of the built environment included 14 different indoor and outdoor PA-related facilities on school grounds. Administrators were asked to report if their "school has access to any of the following PA-related facilities during school hours". Those who reported having the PA-related facility on school grounds (Yes, on grounds) were compared to those who reported not having the PA-related facility on school grounds [Yes, off grounds; No; Don't know (referent)]. Since "gymnasium", "room with cardio and weight equipment", and "playing fields" were reported to be available at all 76 schools and "playground equipment" was not available to high school students at any of the 76 schools, these factors were excluded from the analysis. A school facilities index was also created representing the cumulative number of PA-related facilities available on school grounds on a continuum of 0 to 10, with 1 point on the index awarded for each different type of PA-related facility available on school grounds regardless of the number of facilities available (e.g., 1 point on the index would be awarded to schools having 1, 2, or 4 outdoor hoops) and did not include gymnasiums, room with cardio and weight equipment, or playing fields.

Built environment variables located within a 1-km circular buffer of each school including the density of recreation facilities (includes dance studios, fitness/gym facilities, sport and recreation clubs, and golf courses), parks, fast-food outlets, and shopping malls were recorded. Three measures of neighbourhood design features were also considered, land mix use, residential density, and street connectivity, independently as well as part of a walkability index^a ^[30].

#### School characteristics

An administrator at each school reported the location of the school (rural, urban, suburban). Based on the date of data collection, schools were classified according to the season in which data were collected. As in other studies [27,31], common seasons (winter: December 21-March 20, spring: March 21-June 20, fall: September 21-December 20) were used. Data collected from schools in the winter (referent) were compared to data collected from schools in the spring and fall seasons.

Using data from the 2006 Canadian Census Tract Profiles, the area-level SES measure for each school was based on the proportion of households in the census tract living below the Statistics Canada low-income cut-off (LICO) level. The LICO values identify those who are substantially worse off than the average population as it represents the proportion of households in the census tract that attribute 20% more than the average Canadian family to food, shelter, and clothing [32]. There are different cut-offs according to the number of people in the household and whether the household is located in a rural, suburban, or large urban area. These values are based on after-tax income. The LICO function at the census tract level was available for postal codes of 56 schools (74%) of schools. School postal codes that did not have a LICO value at the census tract level were taken at the level of the census agglomeration (i.e., one level less specific, typically for rural areas).

### Analyses

Due to the hierarchical nature of these data (students nested within schools), a gender-specific hierarchical linear regression modeling approach was used to evaluate the degree to which school characteristics were associated with males' and females' time spent in PA while controlling for student-level and potential school-level confounding variables. Consistent with previous research [33-35], a three-step modeling procedure was used to examine adolescent PA. Step 1 determined the across school variability in students' time spent in PA.

Step 2 included a series of univariate analyses examining if each of the school characteristics was associated with male and female students' time spent in PA. School PA facility and walkability index variables were also examined. In order to not be too restrictive at the initial screening stage, explanatory variables that were not statistically significant (*p *> 0.2) were removed from the analysis.

In Step 3, multivariate models were developed following a blockwise modeling approach. Order of entry into the regression model was based on ecological frameworks positing that multilevel factors influence PA behaviour, from the proximal factors (e.g., student characteristics) to the more distal factors (e.g., school social environment, school and neighbourhood built environment variables). However, only the factors identified as significant in Step 2, were significant at the *p *< .2 level within the block, and contributed to the models were retained in their blocks in the multivariate analysis. Therefore, if all of the variables within a block proved not to significantly contribute to the models the entire block was removed from the analysis. Cross-level interactions with environment-level variables found to be significant in the univariate models and student-level factors were also tested while controlling for potential confounders. Due to their a priori importance grade and weight status were forced into every model regardless of their contribution as well as area-level SES, school location, and season of data collection. Analyses were conducted using PROC MIXED in SAS version 9.2 (Cary, NC).

## Results

### Student characteristics

This study included both males (50.4%, n = 10973) and females (49.6%, n = 10781) across grades 9 to 12 (Table 1). As shown in Table 1, males (mean PA = 166.8 min/day, SD: ± 101.2) reported spending significantly more minutes per day in MVPA than females (mean PA = 134.7 min/day, SD: ± 88.1; t = 24.9, *p *< .0001). Yet, the prevalence of overweight and obesity was significantly higher among males (28.0%, mean BMI = 22.05, SD: ± 3.52) than females (18.1%, mean BMI = 21.34, SD: ± 3.41). More males than females also used active and mixed modes of transportation to school (*X*^2 ^= 178.87, *p *< .0001), enrolled in PE (*X*^2 ^= 100.73, *p *< .0001), participated in school intramurals (*X*^2 ^= 279.63, *p *< .0001) and varsity sports teams (*X*^2 ^= 124.86, *p *< .0001), and engaged in strength training activities three or more days per week (*X*^2 ^= 191.68, *p *< .0001). More females than males, however, engaged in flexibility-related activities three or more days per week (*X*^2 ^= 148.18, *p *< .0001).

**Table 1:** Descriptive statistics for student respondents

Student Characteristic	Females n = 10781[% (n)]	Males N = 10973[% (n)]	Chi-square/t-test (sex differences)
**Grade**			
9	27.3 (2938)	27.6 (3035)	*X*^2 ^= 1.14, *p *< 0.7670
10	26.9 (2902)	26.4 (2890)	
11	23.2 (2499)	23.0 (2521)	
12	22.6 (2442)	23.0 (2527)	
**Body Mass Index (BMI)**			
Underweight	1.3 (145)	1.7 (181)	*X*^2 ^= 77.07, *p *< .0001
Healthy weight	80.6 (8685)	70.3 (7718)	
Overweight	13.3 (1433)	19.8 (2171)	
Obese	4.8 (518)	8.2 (903)	
**Mode of Transportation to School**			
Active	16.8 (1816)	23.7 (2600)	*X*^2 ^= 178.87, *p *< .0001
Mixed	24.6 (2650)	20.2 (2212)	
Inactive	58.6 (6315)	56.1 (6161)	
**Enrolled in school PE**			
Yes	31.4 (3391)	38.0 (4163)	*X*^2 ^= 100.73, *p *< .0001
No	68.6 (7390)	62.0 (6810)	
**Participates in school intramurals**			
Yes	27.9 (3003)	38.2 (4193)	*X*^2 ^= 279.63, *p *< .0001
No	72.1 (7778)	61.8 (6780)	
**Participates in school varsity teams**			
Yes	37.5 (4043)	45.0 (4932)	*X*^2 ^= 124.86, *p *< .0001
No	62.5 (6738)	55.0 (6041)	
**Participates in flexibility activities**			
Less than 3 days per week	40.4 (4357)	48.7 (5341)	*X*^2 ^= 148.18, *p *< .0001
At least 3 days per week	59.6 (6424)	51.3 (5632)	
**Participates in strength training**			
Less than 3 days per week	36.6 (3948)	27.9 (3056)	*X*^2 ^= 191.68, *p *< .0001
At least 3 days per week	63.4 (6833)	72.1 (7917)	
Physical Activity Time*			
Average minutes of moderate to vigorous PA per day	134.7 (88.1)	166.8 (101.2)	t = 24.9, *p *< .0001

*Mean (SD; Range) presented for continuous variable

### Student- and environment-level characteristics associated with female physical activity

Significant between-school variation was identified for female PA [σ_μ0_^2 ^= 7600.26 (163.00)], where school-level differences accounted for 2.1% of the variability in female PA. Building on the results of the univariate analyses in Table 2, findings from the final model shown in Table 3 indicate the school-level variable found to positively associate with females' time spent in PA was attending a school with an alternate room for PA (β = 12.51 (3.96), *p *= 0.0024). Land-use mix diversity (β = -26.14 (10.19), *p *= 0.0124) was the school-level variable found to negatively associate with females' time spent in PA. Student-level variables positively associated with females' time spent in PA included using an active mode of transportation (β = 18.28 (2.23), *p *< .0001), using a mixed mode of transportation (β = 4.00 (1.90), *p *= 0.0354), enrolling in PE (β = 24.10 (1.86), *p *< .0001), participating in school intramurals (β = 18.32 (2.19), *p *< .0001), participating on school varsity teams (β = 9.36 (2.03), *p *< .0001), engaging in flexibility activities at least 3 days per week (β = 26.96 (1.90), *p *< .0001), and engaging in strength activities at least 3 days per week (β = 22.91 (1.96), *p *< .0001). Being in grades 11 (β = -7.77 (2.28), *p *< .0001) and 12 (β = -12.77 (2.34), *p *< .0001; referent = grade 9) were negatively associated with females' time spent in PA. Although not shown in Table 3, the association between female PA and the walkability index (β = -0.74 (0.28), *p *= 0.0096) remained significant even after adjusting for all other variables in the final model.

**Table 2:** School descriptives and univariate analyses examining associations between school characteristics and students' time spent in PA

		Univariate analyses			
	**School Descriptives (N = 76)**	**Females (N = 76, n = 10781)**		**Males (N = 76, n = 10973)**	

**School Characteristic**	**% (N)/Mean (SD; Range)**	**β (SE)**	**p-value**	**β (SE)**	**p-value**

**School curriculum and instruction**					

Offer daily PE	72.4 (55)	**7.82 (3.83)**	**0.0445^a^**	**9.36 (4.95)**	**0.0629^a^**

Offer intramurals	76.3 (58)	-**4.95 (3.98)**	**0.2177^a^**	-4.61 (5.19)	0.3782

Offer interschool sports	86.8 (66)	0.12 (5.38)	0.9816	4.02 (6.96)	0.5652

**School built environment**					

Other room for PA	80.3 (61)	**7.18 (4.48)**	**0.1132^a^**	**8.20 (5.80)**	**0.1613^a^**

Dance studio	36.8 (28)	-1.71 (3.58)	0.6349	-2.82 (4.59)	0.5410

Swimming pool	6.6 (5)	1.42 (7.06)	0.8410	5.47 (8.95)	0.5435

Baseball diamond	36.8 (28)	-1.89 (3.62)	0.6054	-2.87 (4.65)	0.5390

Outdoor hoops	51.3 (39)	-0.77 (3.50)	0.8265	-3.18 (4.47)	0.4795

Tennis court	19.7 (15)	-4.42 (4.31)	0.3089	-2.06 (5.54)	0.7110

Paved area for games	46.1 (35)	1.09 (3.50)	0.7558	-4.27 (4.45)	0.3412

Bicycle racks	82.9 (63)	-2.76 (4.80)	0.5672	-0.67 (6.14)	0.9132

Skating rink	7.9 (6)	5.75 (6.41)	0.3730	2.92 (8.27)	0.7246

Running/walking track	86.8 (66)	-0.16 (5.34)	0.9755	-1.11 (6.75)	0.8693

School PA facilities index	5.4 (1.7, 1-10)	0.10 (1.02)	0.9199	0.68 (1.30)	0.6014

**Neighbourhood built environment**					

Fast food outlets	2.8 (3.5, 0-15)	-0.02 (0.50)	0.9685	-0.26 (0.64)	0.6817

Recreation facilities	1.6 (2.5, 0-13)	-0.55 (0.69)	0.4259	-0.69 (0.89)	0.4451

Shopping malls	0.4 (0.8, 0-4)	2.44 (2.19)	0.2692	2.36 (2.82)	0.4060

Parks	0.6 (1.4, 0-9)	2.13 (3.81)	0.5781	3.97 (4.84)	0.4145

Street connectivity	148.9 (81.3, 0-360.0)	-**0.03 (0.02)**	**0.1634^a^**	-**0.04 (0.03)**	**0.1386^a^**

Land-use mix diversity	0.5 (0.2, 0-0.8)	-**22.66 (10.60)**	**0.0355^a^**	-11.38 (13.76)	0.4107

Residential density	808.2 (778.2, 0.9-3906.0)	0.00 (0.00)	0.6825	0.00 (0.00)	0.2791

Walkability index	0.2 (5.7; -18.8-12,4)	-**0.59 (0.28)**	**0.0352^a^**	-**0.45 (0.36)**	**0.2133^a^**

Note: ^a^*p *< 0.2

**Table 3:** Multilevel regression analysis for female students' time spent in PA (n=10781)

Characteristics		Student Variables	Student and School Social Environment Variables	Student, School Social Environment, and School Built Environment Variables	Student, School Social Environment, and School & Neighbourhood Built Environment Variables	Student, School Social Environment, School & Neighbourhood Built Environment Variables, and School Demographics
		**β (SE)**	**β (SE)**	**β (SE)**	**β (SE)**	**β (SE)**

Individual-level						

Grade	9	REF	REF	REF	REF	REF

	10	-2.11 (2.18)	-2.13 (2.18)	-2.13 (2.18)	-2.15 (2.18)	-2.11 (2.18)

	11	-7.81 (2.28)^c^	-7.81 (2.28)^c^	-7.81 (2.28)^c^	-7.79 (2.28)^c^	-7.77 (2.28)^c^

	12	-13.00 (2.35)^c^	-13.00 (2.35)^c^	-12.94 (2.35)^c^	-12.88 (2.35)^c^	-12.77 (2.34)^c^

Weight status	Healthy weight	REF	REF	REF	REF	REF

	Underweight	10.55 (6.76)	10.54 (6.76)	10.57 (6.76)	10.63 (6.76)	10.86 (6.76)

	Overweight	-0.10 (2.31)	-0.10 (2.31)	-0.10 (2.31)	-0.09 (2.31)	-0.15 (2.31)

	Obese	3.14 (3.68)	3.17 (3.68)	3.23 (3.68)	3.17 (3.68)	3.06 (3.68)

Mode of transport to school	Non-active	REF	REF	REF	REF	REF
	
	Active	18.00 (2.23)^c^	18.02 (2.23)^c^	18.10 (2.23)^c^	18.09 (2.23)^c^	18.28 (2.23)^c^
	
	Mixed	3.72 (1.90)^a^	3.68 (1.90)^a^	3.75 (1.90)^a^	3.85 (1.90)^a^	4.00 (1.90)^a^

Enrolled in PE	No	REF	REF	REF	REF	REF

	Yes	23.87 (1.86)^c^	23.90 (1.86)^c^	23.90 (1.86)^c^	23.94 (1.86)^c^	24.10 (1.86)^c^

Participating in school intramurals	No	REF	REF	REF	REF	REF
	
	Yes	18.36 (2.19)^c^	18.31 (2.19)^c^	18.34 (2.19)^c^	18.31 (2.19)^c^	18.32 (2.19)^c^

Participating in school varsity teams	No	REF	REF	REF	REF	REF
	
	Yes	9.26 (2.03)^c^	9.28 (2.03)^c^	9.32 (2.03)^c^	9.33 (2.03)^c^	9.36 (2.03)^c^

Engaging in flexibility activities	< 3 days/week	REF	REF	REF	REF	REF
	
	≥ 3 days/week	26.90 (1.90)^c^	26.94 (1.90)^c^	26.95 (1.90)^c^	26.93 (1.90)^c^	26.96 (1.90)^c^

Engaging in strength activities	< 3 days/week	REF	REF	REF	REF	REF
	
	≥ 3 days/week	22.96 (1.96)^c^	22.93 (1.96)^c^	22.91 (1.96)^c^	22.95 (1.96)^c^	22.91 (1.96)^c^

Environment-level						

Offer daily PE	No		REF	REF	REF	REF

	Yes		5.26 (3.91)	5.37 (3.79)	6.31 (3.70)	5.68 (3.65)

Offer intramurals	No		REF	REF	REF	REF

	Yes		-5.65 (4.01)	-5.74 (3.88)	-4.26 (3.81)	-6.86 (3.86)

Other room for PA	No			REF	REF	REF

	Yes			9.43 (4.23)^a^	9.46 (4.10)^a^	12.51 (3.96)^b^

Land-use mix diversity					-24.88 (9.93)^b^	-26.14 (10.19)^b^

Street connectivity					*removed to create more parsimonious model	removed to create more parsimonious model

-2LL^d^		125247	125233	125223	125210	125177

Note: ^a^*p *< 0.05; ^b^*p *< 0.01, ^c^*p *< 0.001, ^d^-2LL: -2 Log Likelihood (smaller is better fit); Controlling for potential school-level confounders: area-level SES, school location (rural, suburban, urban), and season of data collection (winter, fall, spring)

**Figure 1:**
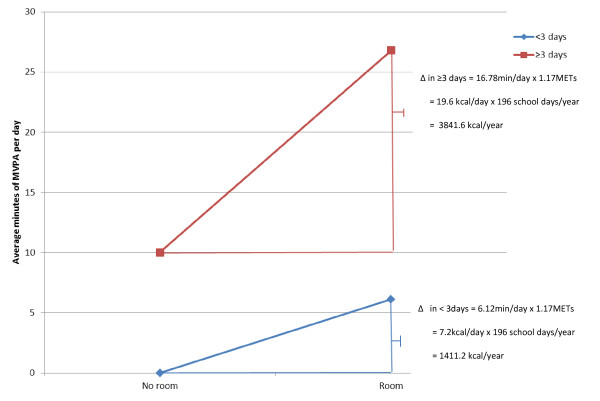
**Contextual Interaction between participation in flexibility-related activities and attending a school with another room for PA**. Using the model estimates, the average minutes of MVPA per day for female adolescents can be estimated as a function of both participating in yoga and stretching activities and attending a school with another room for PA. In Figure 1, the model-based estimates of a female adolescent relative to a hypothetical female adolescent who participates in yoga and stretching activities and attends a school without a another room for PA are presented.

A significant contextual interaction was identified between participating in flexibility-related activities and attending a school with an alternate room for PA (F = 6.13, *p *= 0.013). As shown in Figure 1, females who participated in ≥ 3 days per week of flexibility-related activities engaged in more minutes of PA but the amount of time spent in PA was significantly higher among females attending schools with an alternate room for PA compared to females attending schools without this facility. Using conservative energy expenditure estimates [36], our finding suggests that a female student who participated in ≥ 3 days per week of flexibility activities and attended a school with an alternate room for PA would expend roughly 3840 kcal/school year more than a female student who participated in ≥ 3 days per week of flexibility activities and did not attend a school with an alternate room for PA, about equivalent to the caloric value in one pound of fat.

### Student- and environment-Level characteristics associated with male physical activity

Significant between-school variation was identified for male PA [σ_μ0_^2 ^= 9993.02 (287.74)]. Using the null models, we found school-level differences accounted for 2.8% of the variability in males' time spent in PA when controlling for individual-level variance.

Building on the results of the univariate analyses (Table 2) and using a blockwise modeling approach, findings from the final model shown in Table 4 indicate school-level variables found to positively associate with males' time spent in PA included attending a school with an alternate room for PA (β = 13.32 (5.21), *p *= 0.0127). Student-level variables positively associated with males' time spent in PA included using an active mode of transportation (β = 11.69 (2.37), *p *< .0001), using a mixed mode of transportation (β = 6.41 (2.36), *p *= 0.0065), enrolling in PE (β = 19.83 (1.97), *p *< .0001), participating in school intramurals (β = 23.78 (2.28), *p *< .0001), participating in school varsity teams (β = 12.36 (2.23), *p *< .0001), engaging in flexibility-related activities on at least 3 days per week (β = 23.18 (1.99), *p *< .0001), and engaging in strength activities on at least 3 days per week (β = 33.83 (2.21), *p *< .0001). Student-level variables found to negatively associate with males' time spent in PA included being in grades 11 (β = -10.59 (2.54), *p *< .0001) or 12 (β = -19.86 (2.59), *p *< .0001; referent = grade 9), and being obese (β = -8.63 (3.47), *p *< .0001; referent = healthy weight). Although not shown in Table 4, a separate model including the walkability index and student demographic, school social environment, school built environment, and potential school-level confounding variables was also examined. The association between males' time spent in PA and the walkability index (β = -2.15 (0.9003), *p *= 0.0196) remained significant even after adjusting for all other variables in the final model.

**Table 4:** Multilevel regression analysis for male students' time spent in PA (n = 10973)

Characteristics		Student Variables	Student and School Social Environment Variables	Student, School Social Environment, and School Built Environment Variables	Student, School Social Environment, and School & Neighbourhood Built Environment Variables	Student, School Social Environment, School & Neighbourhood Built Environment Variables, and School Demographics
		**β (SE)**	**β (SE)**	**β (SE)**	**β (SE)**	**β (SE)**

Individual-level						

Grade	9	REF	REF	REF	REF	REF

	10	-4.64 (2.46)	-4.63 (2.46)	-4.69 (2.46)	-4.64 (2.46)	-4.67 (2.46)

	11	-10.60 (2.54)^c^	-10.61 (2.54)^c^	-10.63 (2.54)^c^	-10.61 (2.54)^c^	-10.59 (2.54)^c^

	12	-19.96 (2.59)^c^	-20.00 (2.59)^c^	-20.02 (2.59)^c^	-20.02 (2.59)^c^	-19.86 (2.59)^c^

Weight status	Healthy weight	REF	REF	REF	REF	REF

	Underweight	4.20 (7.02)	4.26 (7.02)	4.33 (7.02)	4.32 (7.02)	4.38 (7.02)

	Overweight	-3.70 (2.26)	-3.71 (2.26)	-3.71 (2.26)	-3.71 (2.26)	-3.74 (2.26)

	Obese	-8.73 (3.29)^c^	-8.72 (3.29)^b^	-8.77 (3.29)^b^	-8.78 (3.29)^b^	-8.63 (3.29)^b^

Mode of transport to school	Non-active	REF	REF	REF	REF	REF
	
	Active	11.48 (2.29)^c^	11.39 (2.29)^c^	11.46 (2.29)^c^	11.70 (2.29)^c^	11.69 (2.37)^c^
	
	Mixed	6.20 (2.36)^b^	6.12 (2.36)^b^	6.18 (2.35)^b^	6.37 (2.36)^b^	6.41 (2.36)^b^

Enrolled in PE	No	REF	REF	REF	REF	REF

	Yes	19.80 (1.97)^c^	19.76 (1.97)^c^	19.78 (1.97)^c^	19.76 (1.97)^c^	19.83 (1.97)^c^

Participation in school intramurals	No	REF	REF	REF	REF	REF
	
	Yes	23.80 (2.28)^c^	23.77 (2.28)^c^	23.81 (2.28)^c^	23.79 (2.23)^c^	23.78 (2.28)^c^

Participation in school varsity teams	No	REF	REF	REF	REF	REF
	
	Yes	12.38 (2.23)^c^	12.39 (2.23)^c^	12.41 (2.23)^c^	12.41 (2.23)^c^	12.36 (2.23)^c^

Engaging in flexibility activities	< 3 days/week	REF	REF	REF	REF	REF
	
	≥ 3 days/week	23.10 (1.99)^c^	23.10 (1.99)^c^	23.13 (1.99)^c^	23.11 (1.99)^c^	23.18 (1.99)^c^

Engaging in strength activities	< 3 days/week	REF	REF	REF	REF	REF
	
	≥ 3 days/week	33.84 (2.21)^c^	33.87 (2.21)^c^	33.84 (2.21)^c^	33.85 (2.21)^c^	33.83 (2.21)^c^

Environment-level						

Offering daily PE	No		REF	REF	REF	REF

	Yes		9.28 (4.91)	9.52 (4.77)^a^	9.19 (4.72)	7.65 (4.50)

Other room for PA	No			REF	REF	REF

	Yes			11.66 (5.55)^a^	10.47 (5.54)	13.32 (5.21)^b^

Street connectivity					-0.04 (0.03)	-0.03 (0.03)

-2LL^d^		130638	130626	130616	130619	130579

Note: ^a^*p *< 0.05; ^b^*p *< 0.01, ^c^*p *< 0.001, ^d^-2LL: -2 Log Likelihood (smaller is better fit); Controlling for potential school-level confounders: area-level SES, school location (rural, suburban, urban), and season of data collection (winter, fall, spring)

**Figure 2:**
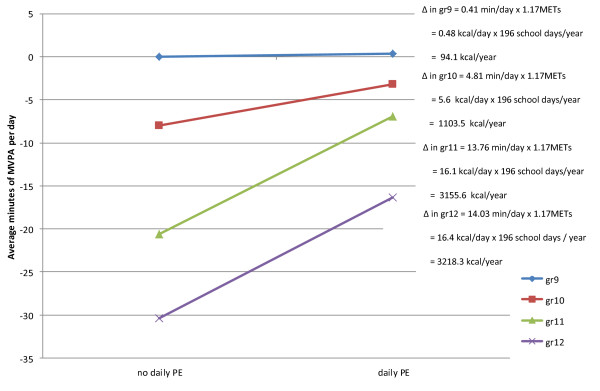
**Contextual Interaction between grade and attending a school offering daily PE**. Using the model estimates, the average minutes of MVPA per day for male students can be estimated as a function of both grade and attending a school offering PE daily (5 days/week). In Figure 2, the model-based estimates of a male student relative to a hypothetical male student who is grade 9 and attends a school that does not offer PE daily are presented.

A significant interaction was also detected for male PA between grade 11 (F = 5.22, *p *= 0.0224) and grade 12 (F = 5.24, *p *= 0.0221) and attending a school that offers daily PE. This interaction indicates the relationship between male PA and grade level is significantly different for males attending schools that do and do not offer daily PE (Figure 2). Figure 2 illustrates males who are in grades 11 and 12 engaged in less minutes of PA than their grade 9 counterparts but the decrease was significantly lower among males attending schools that offer daily PE compared to schools that do not offer daily PE. Using conservative estimates [36], our finding suggests that males in grades 11 and 12 who attended a school that offers daily PE expend roughly 3155.6 kcal/school year and 3218.3 kcal/school year more than males in grades 11 and 12 who did not attend a school that offers daily PE, about equivalent to the caloric value in one pound of fat.

## Discussion

Gender-specific investigations in PA research are critical due to the well-established gender discrepancies in PA among youth and the need to inform gender-focused interventions for PA [3,6,37]. Findings from the present study revealed significant differences in the time males and females spend in PA as well as in some of the school- and student-level factors associated with PA.

### Importance of school environment

Consistent with the tenants of Ecological Theory [38], we identified that school-level differences accounted for a significant amount of the variability in females (2.1%) and males' (2.8%) time spent in PA suggesting that the characteristics of the school environment a student attends are associated with their PA. These findings are consistent with previous empirical research which suggests that characteristics of the school a student attends can have important impact on their time spent in PA [39,40], even though these earlier studies did not explore the between school variability in PA by gender. Although the amount of and difference between school-level variability in female and male students' time spent in PA in this study appears modest, it is important as even small changes on large numbers of individuals can have appreciable effects [41] and may help account for gender differences in the time spent in PA.

The school-level factor, attending a school with an alternate room for PA, was found to associate with more time spent in PA among both male and female students. To better understand the relationship between attending a school with an alternate room for PA and student PA, the two-way interaction between an alternate room for PA and student-level factors was examined for each gender. An interaction between attending a school with an alternate room for PA and participation in flexibility activities ≥ 3 days per week was significant among females. Females who participated in flexibility activities ≥ 3 days per week reported spending more time in PA especially if they attended a school with an alternate room for PA. Previous research suggests female secondary school students prefer more individual and cooperative activities such as dance and yoga [42]. The lack of indoor space within schools is often cited as a reason for not offering school PE or school PA-related programming [43,44]; therefore, adapting a room for PA within a school may provide the extra space needed to enable additional school PE classes or school PA-programming activities that are known to be particularly attractive to females.

A two-way interaction was also identified between attending a school that offered school PE daily and grade level among males. Consistent with previous research, there was an inverse association between grade level and time spent in PA among students [15,45]; however, our findings indicate males in grades 11 and 12 participated in significantly more minutes of PA if they attended a school that offered daily PE. Although earlier research has shown that students are more likely to enrol in PE if they attend a school that offers daily PE [28], this is the first study to our knowledge, to suggest that the decline in male PA with increasing grade level is attenuated when a male attends a school that offers daily PE. While future research might identify the particular mechanism at work among older students PA participation, school initiatives that seek to encourage PA among male secondary school students might consider offering daily school PE in an effort to increase enrolment in PE and participation in PA.

Finally, walkability was found to negatively associate with both males' and females' time spent in PA. This finding is opposite to the results found for adults in international studies, showing consistently that adults living in in high-walkable neighbourhoods are more physically active [46,47]. According to Van Dyck and colleagues (2009) [48], who also found a negative relationship between PA and walkability among a sample of Belgian adolescents, this suggests that the associations between neighbourhood walkability and PA may be different for adolescents than for adults, which is important for the development of future environmental interventions.

### Importance of student characteristics

One notable gender discrepancy between the student level factors associated with students' time spent in PA is the negative association with weight status. Being obese had a strong negative association with males' time spent in PA, whereas there was no discernable pattern in the association between females' weight status and time spent in PA. Although atypical, other studies have also found similar gender differences in the relationship between weight status and PA among youth where overweight and obese females are not significantly less active than normal weight females [3,45,49]. A possible explanation for this result is that male weight status appears to be more related to PA participation than to other obesity-related behaviours such as eating or sedentary (e.g., television or video game) habits [6,50]. Alternatively, this finding could reflect the tendency for overweight females to over-report PA compared to normal weight females [51]. The finding may also be a function of missing data, as previous research has identified that respondents with missing BMI data were more likely to be female and to have daily energy expenditure values than those children with BMI data [52]. Given the findings of this study indicate obese males are particularly vulnerable to inactivity, school PA promotion initiatives should consider targeting this population.

Of the school PE and PA-related activities examined in this study, enrolment in PE and participation in flexibility activities were the only two that had a stronger positive effect on females' time spent in PA compared with males. School intramurals and varsity sports teams often include traditional competitive sports-related activities that have been reported to be more attractive to males than females [43,53,54]. Indeed, research suggests females typically enjoy more individual, cooperative, and recreation activities, such as yoga, dance and aerobics [43,55]. Secondary school PE classes in Ontario do offer female-only classes as well as curriculum that can cater to the PA needs and interests of females if implemented by PE teachers appropriately. Designing PE classes to support participation and encourage success among females by incorporating flexibility activities and other female-friendly programming should be considered.

### Limitations

This study is subject to some limitations. Causal relationships cannot be inferred from these cross-sectional data; however, the relationship is beneficial for understanding associations. Over-reporting of PA and under-reporting or missing BMI data is always possible with self-report instruments; however, the SHAPES student-level PA module has been validated against objectively measured PA, height, and weight [20]. Also, the 1-km school buffer zone may not have provided a complete picture of the PA opportunity structures accessible to secondary students, who may have access to transportation taking them beyond the buffer. Moreover, the availability of churches in the school neighbourhood was not considered as a feature of the school built environment despite recent evidence indicating a positive association with adolescent female PA [17]. Finally, the study involved secondary data analysis so data were not available for all of the measures in an ideal study. For example, individual-level measures of SES (e.g., household SES) were not available and area-level SES measures have been found to be weaker predictors of adolescent PA in Canada by comparison to individual SES measures [56].

## Conclusions

Because of the significant discrepancy in the time spent in PA among males and females, and some school- and student-level variables associated with students' time spent in PA are not consistent across genders, interventions promoting PA should take gender differences into account. Results demonstrate school variation exists for both male and female students indicating the school a student attends influences the students' time spent in PA over and above student characteristics. To increase student PA, schools should consider providing an alternate room for PA, especially for flexibility activity programming among females. Schools should also consider offering daily PE classes to male students in senior grades and providing PA promotion initiatives specifically targeting obese males. Additional studies examining gender differentials in the associations between factors of the school environment and student PA are warranted. Experimental studies with random allocation of change and no change to features of the school environment or natural experiments testing male and female PA pre- and post-change to features of the school environment would be stronger research designs to test the effect of the school environment on student PA, but less feasible.

## Endnotes

^aWalkability index= (6 x z-score land-use mix diversity) + (z-score street connectivity) + (z-score residential density) ^

## Competing interests

The authors declare that they have no competing interests.

## Authors' contributions

EH carried out statistical analysis and drafted the manuscript. SL and SM contributed to study design, interpretation of the data, and revising the manuscript. SL and SM were the principal investigators of the larger SHAPES-Ontario project and responsible for the project design, Organization, and implementation. All authors read and approved the final manuscript.

## Pre-publication history

The pre-publication history for this paper can be accessed here:

http://www.biomedcentral.com/1471-2458/12/74/prepub
